# Association of TNF-α, TGF-β1, IFN-γ, IL-6, and IL-10 Gene Polymorphisms with Hodgkin Lymphoma: Susceptibility and Clinical Outcomes

**DOI:** 10.3390/jcm15187210

**Published:** 2026-09-17

**Authors:** Handan Haydaroglu Sahin, Duygu Deniz Ustul, Pelin Yorulmaz

**Affiliations:** 1 Division of Hematology, Gaziantep University Hospital, Gaziantep 27310, Türkiye; 2 Division of Internal Medicine, Kusadası Government Hospital, Aydın 09400, Türkiye; duygu.denizzz9@gmail.com; 3 Division of Internal Medicine, Gaziantep City Hospital, Gaziantep 27470, Türkiye; drpelinacel@gmail.com

**Keywords:** cytokine gene polymorphism, Hodgkin lymphoma, susceptibility, progression-free survival, genetic association

## Abstract

**Background:** In Hodgkin lymphoma (HL), cytokine-related genetic variation may influence disease susceptibility and clinical outcomes. This study investigated polymorphisms in tumor necrosis factor-alpha (TNF-α), transforming growth factor-beta 1 (TGF-β1), interferon-gamma (IFN-γ), interleukin-6 (IL-6), and interleukin-10 (IL-10) in relation to HL susceptibility and progression-free survival (PFS). **Methods:** Seventy patients with HL and 70 healthy controls were included. Cytokine gene polymorphisms were analyzed from peripheral blood using sequence-specific primer polymerase chain reaction (PCR-SSP). Multiple comparisons were controlled using the Benjamini–Hochberg false discovery rate (FDR), and prognostic associations were evaluated using Firth penalized Cox regression. **Results:** Several genotype-level associations were nominally significant before multiple-testing correction, including TNF-α (−308) GA, IFN-γ (+874) TT, IL-6 (−174) GG, TGF-β1 codon 10 TT, IL-10 (−1082) GG, IL-10 (−819) CC, and IL-10 (−592) CC genotypes. In addition, selected TGF-β1 and IL-10 predefined multilocus genotype combinations showed nominal associations with HL susceptibility. However, none of these genotype-level or multilocus genotype-combination associations remained statistically significant after FDR correction. In contrast, significant allele-level associations persisted after FDR adjustment for the IL-6 (−174) G allele, TGF-β1 codon 10 T allele, and IL-10 (−1082) G, IL-10 (−819) C, and IL-10 (−592) C alleles. Bone marrow involvement remained associated with inferior PFS (HR = 11.62, 95% confidence interval (CI): 3.61–34.66; *p* < 0.001), although the wide CI warrants cautious interpretation. The IL-6 (−174) CC genotype also showed an association with inferior PFS (HR = 7.09, 95% CI: 1.34–25.29; *p* = 0.025); however, this estimate was based on only two carriers and should be considered exploratory. **Conclusions:** Selected cytokine gene variants, particularly at the allele level, may be associated with HL susceptibility. The prognostic association of IL-6 (−174) CC requires confirmation in larger cohorts.

## 1. Introduction

Recent population-based data indicate changing incidence and increasing prevalence of Hodgkin lymphoma (HL), with variation across age and sex groups [1]. Although its etiology remains incompletely understood, viral infections, immunological alterations, familial predisposition, socioeconomic factors, and somatic genetic mutations have been implicated in its pathogenesis [2].

Cytokines produced by Hodgkin/Reed–Sternberg (HRS) cells and surrounding inflammatory cells play an important role in the pathobiology and clinical manifestations of HL. Interleukin (IL)-2, IL-5, IL-6, IL-7, IL-9, IL-10, granulocyte–macrophage colony-stimulating factor, lymphotoxin-α, and transforming growth factor (TGF-β) are among the cytokines secreted by HRS cells and contribute to HRS-cell survival and proliferation, inhibition of apoptosis, and the characteristic clinicopathological features of HL. Specific associations have been reported between IL-5 and tissue eosinophilia, IL-10 and EBV-positive HL, TGF-β and fibrosis, and tumor necrosis factor-alpha (TNF-α)/TNF-β and weight loss [3,4]. Furthermore, cytokines produced by HRS cells may contribute to HL pathogenesis by acting as autocrine growth factors [5]. Among these, IL-2, IL-6, IL-10, IL-13, and the proinflammatory cytokine TNF-α have been implicated in tumor-promoting processes, including proliferation, angiogenesis, and resistance to apoptosis [4].

Given the central role of cytokines in HL biology, genetic variants previously associated with altered cytokine production may influence disease susceptibility and clinical outcomes. However, studies investigating cytokine-related genetic variation in HL are limited, and their findings have been inconsistent. Previous studies have evaluated IL-10 (−1082, −819, and −592) [6,7], IL-6 (−174) [7,8], TNF-α (−308) [6,7], and Interferon-gamma (IFN-γ) (+874) [7] in relation to HL susceptibility and/or clinical outcomes, with conflicting results across different study populations. TGF-β1 codon 10 and codon 25 variants were additionally selected based on their reported functional relevance and previous investigation in hematological malignancies [9,10]. Based on these previous reports, we selected these regions to further evaluate their potential associations with HL susceptibility and clinical outcomes in our patient population.

Accordingly, this study aimed to evaluate selected polymorphisms in five cytokine genes—TNF-α, TGF-β1, IFN-γ, IL-6, and IL-10—and their associations with HL susceptibility and clinical outcomes.

## 2. Materials and Methods

### 2.1. Study Subjects

Adult patients (≥18 years) with Hodgkin lymphoma (HL) who were diagnosed between 1 April 2002 and October 2019 and treated and followed at the Hematology Clinic of Gaziantep University Faculty of Medicine were retrospectively identified from institutional medical records. Patients were eligible if they had histopathologically confirmed HL, received first-line treatment with curative intent, and had sufficient clinical and follow-up data available for analysis. Patients who did not receive treatment, had nodular lymphocyte-predominant Hodgkin lymphoma, or had incomplete key clinical or laboratory data were excluded. Clinical and outcome data were retrospectively updated through 30 October 2020, which served as the cutoff date for follow-up analyses.

The diagnosis of HL was histopathologically confirmed by examination of tissue biopsy specimens, with identification of characteristic HRS cells in an appropriate cellular background. Histological subtypes were classified according to the World Health Organization (WHO) classification. At diagnosis, patients underwent medical history and physical examination, including assessment of B symptoms and lymph node, liver, and spleen involvement. Baseline laboratory evaluation included complete blood count, erythrocyte sedimentation rate (ESR), C-reactive protein (CRP), β2-microglobulin, albumin, and lactate dehydrogenase (LDH). Disease extent was assessed using 18F-fluorodeoxyglucose positron emission tomography/computed tomography (18F-FDG PET/CT) and/or contrast-enhanced computed tomography of the chest, abdomen, and pelvis; bone marrow evaluation was performed when clinically indicated.

The control group consisted of healthy individuals aged ≥18 years from the same ethnic and geographic population as the patient group, without known comorbidities. Individuals with active infection or a history of malignancy were excluded. The control group was selected to have an age and sex distribution comparable to that of the patient group. At our institution, written informed consent permitting the use of biological specimens and corresponding clinical records for scientific research is routinely obtained at the time of diagnostic sample collection. Accordingly, for patients diagnosed before the ethics approval date, archived DNA obtained from diagnostic blood samples was used under this prior written consent for research use. Written informed consent was also obtained from the healthy controls enrolled in the present study. The study was approved by the Gaziantep University Faculty of Medicine Ethics Committee (Decision No. 2019/282; 17 July 2019) and was supported by the Gaziantep University Scientific Research Projects Fund.

Disease stage was determined according to the Ann Arbor staging system. Patients with stage I–II disease were classified as having early-stage disease, whereas those with stage III–IV disease were classified as having advanced-stage disease [11]. In patients with early-stage disease, prognostic risk was assessed according to the European Organisation for Research and Treatment of Cancer (EORTC) criteria, and patients were categorized into favorable- and unfavorable-risk groups [12]. In patients with advanced-stage disease, prognostic assessment was performed using the seven-factor International Prognostic Score (IPS-7) [13].

Progression-free survival (PFS) was defined as the time from diagnosis to disease progression, relapse, or death from any cause; patients without an event were censored at the date of their last documented clinical follow-up or disease assessment.

### 2.2. Treatment and Response Assessment

All patients received doxorubicin, bleomycin, vinblastine, and dacarbazine (ABVD) as first-line treatment. Patients with early-stage disease received two initial cycles of ABVD, followed by response assessment with 18F-FDG PET/CT. Patients with an adequate response continued ABVD, generally to a total of four cycles, whereas treatment was intensified to a BEACOPP-based regimen in patients with an inadequate response when clinically indicated. Patients with advanced-stage disease received ABVD as first-line treatment, generally for a total of six cycles, with treatment modification to a BEACOPP-based regimen in cases of inadequate response. Radiotherapy was administered as consolidation therapy in five patients with bulky disease. Patients with primary refractory or relapsed disease received salvage chemotherapy, including dexamethasone, high-dose cytarabine, and cisplatin (DHAP); gemcitabine and oxaliplatin (GEMOX); or ifosfamide, carboplatin, and etoposide (ICE), followed by autologous hematopoietic stem cell transplantation when eligible. Treatment response was assessed according to the Lugano response criteria using 18F-FDG PET/CT and the Deauville five-point scale. Deauville scores of 1–3 were considered consistent with complete metabolic response, whereas scores of 4–5 were interpreted in conjunction with changes in FDG uptake compared with baseline and the presence or absence of new lesions. Disease progression or relapse was determined based on clinical assessment and 18F-FDG PET/CT findings according to the Lugano response criteria, with biopsy confirmation performed when clinically indicated.

### 2.3. DNA Isolation and Genotyping by the PCR-SSP Method

Peripheral blood samples were collected in EDTA-containing tubes from patients and healthy controls. In the patient group, samples were obtained at diagnosis. For patients diagnosed in earlier years, archived genomic DNA obtained from samples collected at diagnosis was used for genotyping. Genomic DNA was isolated using the High Pure PCR Template Preparation Kit (Roche, Penzberg, Germany; Cat. No. 11796828001) and stored at −80 °C until genetic analysis. Eight polymorphic regions in five cytokine genes were genotyped using the Cytokine Genotyping Tray (One Lambda, Inc., Canoga Park, CA, USA; Cat. No. CYTGEN) according to the manufacturer’s instructions [14]. The analyzed polymorphisms were TNF-α −308 (rs1800629), IFN-γ +874 (rs2430561), IL-6 −174 (rs1800795), TGF-β1 codon 10 (rs1800470; formerly rs1982073) and codon 25 (rs1800471), and IL-10 −1082 (rs1800896), −819 (rs1800871), and −592 (rs1800872). Following agarose gel electrophoresis, reactions showing both the internal-control band and the allele-specific band were interpreted as positive, whereas reactions showing only the internal-control band were interpreted as negative. Samples without an internal-control band were re-amplified.

Predefined multilocus genotype combinations for TGF-β1 codons 10 and 25 and IL-10 −1082, −819, and −592 were evaluated directly from the PCR-SSP genotype patterns according to the manufacturer-provided Cytokine Genotyping Tray evaluation form.

### 2.4. Statistical Analysis

Continuous variables were summarized as median and interquartile range (Q1–Q3), whereas categorical variables were presented as numbers and percentages. No inferential comparisons were performed on continuous variables in their original scale; where applicable, clinically defined categorical cutoffs were used for the analyses.

Genotype distributions and allele frequencies were compared between patients with HL and healthy controls using Fisher’s exact test for 2 × 2 tables and the Fisher–Freeman–Halton exact test for larger contingency tables, as appropriate. Odds ratios (ORs) with 95% confidence intervals (CIs) were calculated for genotypes, alleles, and predefined multilocus genotype combinations. Hardy–Weinberg equilibrium (HWE) was assessed separately in patients and controls using the DeFinetti program. Multiple comparisons were addressed using the Benjamini–Hochberg false discovery rate (FDR) procedure within three predefined hypothesis families: genotype comparisons (23 tests), allele-frequency comparisons (8 locus-level tests), and predefined multilocus genotype-combination comparisons (6 tests). Both raw and FDR-adjusted *p*-values were reported, with FDR-adjusted *p* < 0.05 considered statistically significant for susceptibility analyses.

PFS was estimated using the Kaplan–Meier method, and groups were compared using the log-rank test. Because of the limited number of PFS events and sparse data, multivariable analysis was performed using Firth penalized Cox regression including bone marrow involvement and the IL-6 (−174) CC genotype. Hazard ratios (HRs) with 95% CIs were reported. The proportional hazards assumption was assessed using time-dependent interactions with log(time) and a global test. The analysis was performed using R version 4.5.0 (R Foundation for Statistical Computing, Vienna, Austria).

Other statistical analyses were performed using IBM SPSS Statistics for Windows, version 22.0 (IBM Corp., Armonk, NY, USA). All tests were two-sided; for analyses other than the FDR-adjusted susceptibility comparisons, *p* < 0.05 was considered statistically significant.

## 3. Results

### 3.1. Patient Characteristics

A total of 70 patients with HL and 70 healthy controls were included in the study. Among the patients, 43 (61.4%) were male and 27 (38.6%) were female, with a median age of 32.5 years (Q1–Q3: 26.0–41.5 years). The control group comprised 41 (58.6%) males and 29 (41.4%) females. According to the Ann Arbor staging system, 32 patients (45.7%) had early-stage disease and 38 (54.3%) had advanced-stage disease. During follow-up, disease progression occurred in 20 patients (28.6%). Bone marrow involvement and bulky disease were present in 6 (8.6%) and 5 (7.1%) patients, respectively. Among the 32 patients with early-stage HL, EORTC risk stratification classified 15 (46.9%) as favorable risk and 17 (53.1%) as unfavorable risk. Among the 38 patients with advanced-stage HL, IPS-7 classified 20 (52.6%) as low risk, 16 (42.1%) as moderate risk, and 2 (5.3%) as high risk. The demographic and clinical characteristics of the patients are summarized in Table 1.

### 3.2. Genotyping Analysis

The distributions of TNF-α (−308), IFN-γ (+874), IL-6 (−174), TGF-β1 (codons 10 and 25), and IL-10 (−1082, −819, and −592) gene polymorphisms in patients with HL and healthy controls are summarized in Table 2.

Genotype-Level Associations

Several genotype-level associations were observed at the nominal significance level before correction for multiple comparisons. The TNF-α (−308) GA, IFN-γ (+874) TT, IL-6 (−174) GG, TGF-β1 codon 10 TT, IL-10 (−1082) GG, IL-10 (−819) CC, and IL-10 (−592) CC genotypes were more frequent in patients with HL than in healthy controls (raw *p* < 0.05). However, none of these genotype-level associations remained statistically significant after FDR correction.

Allele-Level Associations

In contrast, several allele-level associations persisted after FDR adjustment. The IL-6 (−174) G allele was more frequent in patients with HL (87.14% vs. 76.43%; OR = 2.09, 95% CI: 1.11–3.93; FDR-adjusted *p* = 0.0483). Similarly, the TGF-β1 codon 10 T allele (73.57% vs. 60.00%; OR = 1.86, 95% CI: 1.12–3.08; FDR-adjusted *p* = 0.0464), IL-10 (−1082) G allele (57.14% vs. 42.85%; OR = 1.78, 95% CI: 1.11–2.85; FDR-adjusted *p* = 0.0464), IL-10 (−819) C allele (86.43% vs. 74.29%; OR = 2.20, 95% CI: 1.19–4.08; FDR-adjusted *p* = 0.0464), and IL-10 (−592) C allele (86.43% vs. 74.29%; OR = 2.20, 95% CI: 1.19–4.08; FDR-adjusted *p* = 0.0464) remained significantly associated with HL. No significant genotype- or allele-level association was observed for TGF-β1 codon 25 after FDR adjustment.

Predefined Multilocus Genotype-Combination Associations

Among the predefined multilocus genotype combinations, the TGF-β1 TC/GC, CC/GG, and TT/GC group and the IL-10 GCC/GCC combination showed nominal associations with HL (raw *p* = 0.0161 and *p* = 0.0299, respectively), but neither remained significant after FDR correction (FDR-adjusted *p* = 0.0897 for both). Genotype distributions deviated from Hardy–Weinberg equilibrium in the HL group for TNF-α (−308) and IL-10 (−1082), whereas the remaining loci were consistent with HWE.

### 3.3. Survival

**Figure 1:**
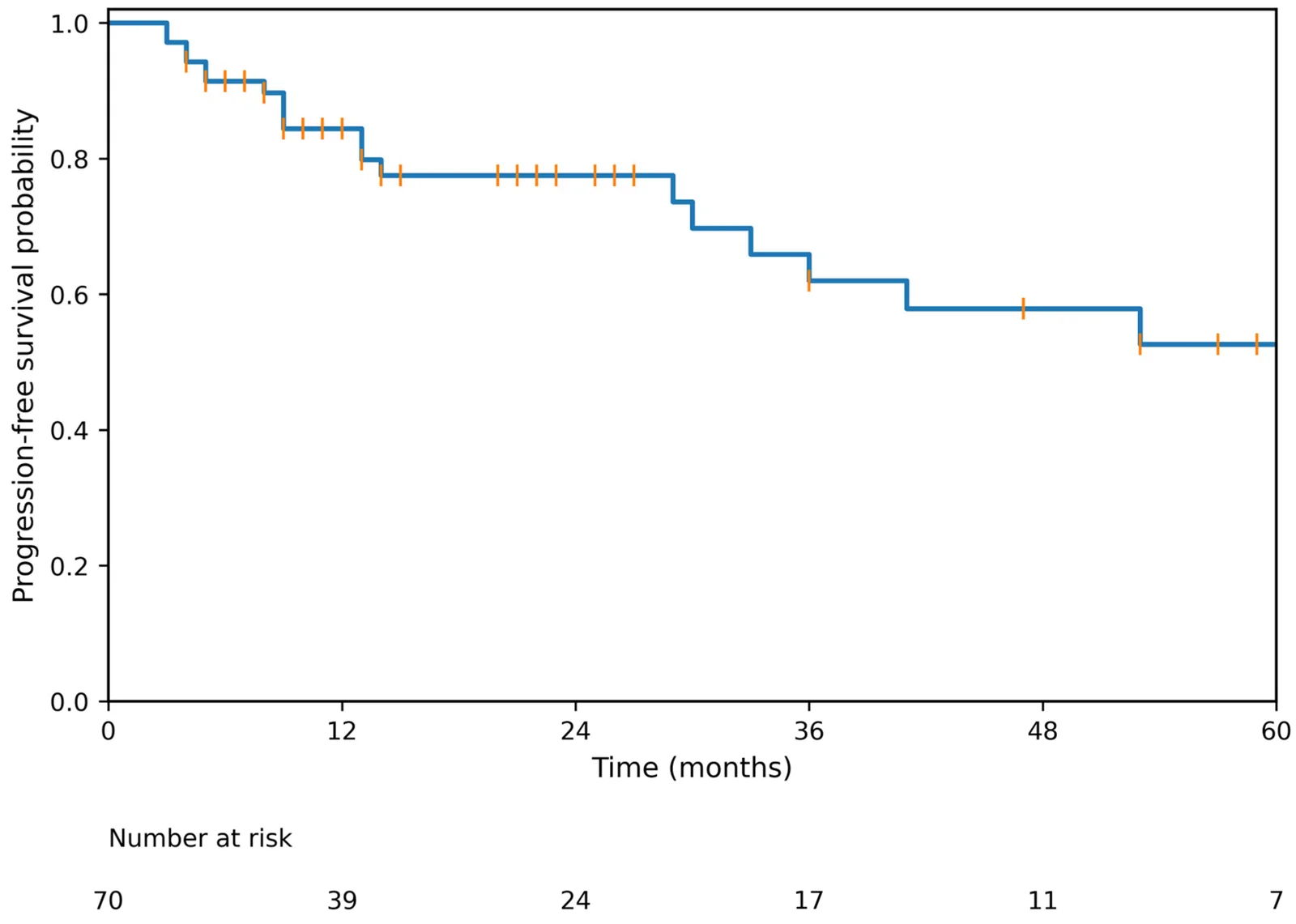
Kaplan–Meier curve for progression-free survival (PFS) in the overall Hodgkin lymphoma cohort. Tick marks indicate censored observations. The number of patients at risk at each time point is shown below the *x*-axis.

A total of 70 patients with HL were included in the survival analysis. During a median follow-up of 19.5 months (range, 4–211 months), 20 patients (28.6%) experienced a PFS event. PFS was estimated using the Kaplan–Meier method. The estimated 5-year PFS for the overall cohort was 52.6% (95% CI: 33.1–68.9%); however, only seven patients remained at risk at 60 months, while 44 patients had been censored before this time point. Therefore, the 5-year estimate should be interpreted cautiously. The number of patients at risk over time is shown in Figure 1.

The results of the univariate log-rank analyses evaluating the associations of clinical prognostic factors and cytokine gene polymorphisms with PFS are presented in Table 3.

In the univariate log-rank analysis, bone marrow involvement (*p* < 0.001) and the IL-6 (−174) CC genotype (*p* = 0.0284) were associated with inferior PFS (Table 3). No other clinical or genetic variables evaluated were significantly associated with PFS (all *p* > 0.05).

In the multivariable Firth penalized Cox regression analysis, bone marrow involvement remained associated with inferior PFS (HR = 11.62, 95% CI: 3.61–34.66; *p* < 0.001). However, because bone marrow involvement was present in only six patients and the confidence interval was wide, the magnitude of this association should be interpreted cautiously. The IL-6 (−174) CC genotype also showed an association with inferior PFS (HR = 7.09, 95% CI: 1.34–25.29; *p* = 0.025); however, because this estimate was based on only two CC carriers, it should be considered exploratory and interpreted with caution (Table 4). No evidence of a violation of the proportional hazards assumption was observed (bone marrow involvement × log(time), *p* = 0.943; IL-6 (−174) CC × log(time), *p* = 0.473; global test, *p* = 0.772).

## 4. Discussion

Although Hodgkin lymphoma (HL) is considered a highly curable hematologic malignancy, relapse remains an important clinical challenge, occurring in approximately 15% of high-risk patients with early-stage disease and in up to 40% of those with advanced-stage disease, while approximately 10% develop primary refractory disease [15,16]. Cytokines produced by HRS cells and inflammatory cells in the tumor microenvironment contribute to HL biology and immune regulation and may influence treatment response. Common single-nucleotide polymorphisms (SNPs), particularly in promoter regions, have been associated with altered cytokine production and may thereby influence disease susceptibility and clinical course [6].

IL-10 is an anti-inflammatory cytokine that suppresses Th1-type responses and promotes Th2-type responses. IL-10 polymorphisms have been implicated in susceptibility to infectious and autoimmune diseases and in disease prognosis [17], and have also been associated with acute graft-versus-host disease, transplant-related mortality, and survival after allogeneic transplantation [18]. Several studies have linked IL-10 polymorphisms to NHL risk [19,20], whereas data in HL remain limited and conflicting [6,7,21]. In HL, Munro et al. found no significant association of the IL-10 −1082 or −592 variants with disease susceptibility [21]. Data specifically addressing allele-level associations of IL-10 −1082, −819, and −592 with HL susceptibility remain limited. The IL-10 (−1082) AA genotype has been associated with poorer clinical outcome in DLBCL [19]. In HL, Hohaus et al. reported inferior treatment outcome for the IL-10 −592 AA genotype [6], whereas Schoof et al. identified inferior freedom from treatment failure in carriers of the IL-10 −597 AA, −824 TT, and −1087 AA genotypes [22]. In our study, the IL-10 (−1082) GG, IL-10 (−819) CC, and IL-10 (−592) CC genotypes were more frequent in patients with HL at the nominal significance level; however, these genotype-level associations did not remain statistically significant after FDR correction. At the allele level, the IL-10 (−1082) G, IL-10 (−819) C, and IL-10 (−592) C alleles remained significantly more frequent in patients with HL after FDR adjustment, supporting a potential association with HL susceptibility in our cohort, whereas the corresponding alternative alleles showed inverse associations. Given that the relationship between individual IL-10 variants and cytokine production remains inconsistent, previous studies have also examined haplotype-based associations [6]. Kurreeman et al. demonstrated allele-specific transcription of IL10 and showed that IL10 haplotypes may influence cytokine production [23], whereas Kilpinen et al. reported an association between the ATA haplotype and higher plasma IL-10 levels in healthy individuals [24]. Schoof et al. found that the IL-10 (−1087) G/(−824) C/(−597) C haplotype was associated with better freedom from treatment failure [22]. In the present study, the GCC/GCC predefined multilocus genotype combination was more frequent in patients with HL at the nominal significance level, but this association did not remain statistically significant after FDR correction. Neither the evaluated IL-10 polymorphisms nor the predefined multilocus genotype combinations were significantly associated with PFS in our cohort.

IL-6 is a key regulator of immune responses, cell proliferation, and differentiation [25] and has been implicated in tumor development, invasion, and metastasis [26]. In HL, increased IL-6 expression has been associated with poor treatment response and survival [27]. Hohaus et al. reported less favorable outcomes in patients with the IL-6 (−174) GG genotype [6], whereas Cozen et al. reported that HL risk decreased with an increasing number of IL-6 (−174) C alleles, supporting a potentially protective role of the C allele [8]; Cordano et al. observed a similar trend in young adults, although it was not statistically significant [28]. In our cohort, the IL-6 (−174) G allele remained significantly more frequent in patients with HL after FDR adjustment, supporting a potential association with HL susceptibility, whereas the C allele showed an inverse association. The GG genotype was also more frequent in patients with HL at the nominal significance level; however, this genotype-level association did not remain significant after FDR correction. In the survival analysis, the IL-6 (−174) CC genotype showed an association with inferior PFS in the Firth penalized Cox model. However, because this estimate was based on only two CC carriers, this finding should be considered exploratory rather than evidence of a definitive prognostic factor and should be interpreted with caution.

TNF-α is a major pro-inflammatory cytokine involved in immune regulation, inflammation, the Th1/Th2 balance, and tumor biology [29]. Elevated TNF-α levels have been reported in several malignancies, including lymphomas [30]. The TNF-α (−308) G > A variant has been associated with altered transcriptional activity [31], and TNF-α has an established role in inflammation and tumor biology [29]. Studies have also linked this variant to NHL risk [32,33]. In HL, however, evidence is limited. Hohaus et al. found no significant association between TNF-α (−308, −863) genotypes and HL survival [6]. In our study, the TNF-α (−308) GA genotype was more frequent in patients with HL at the nominal significance level; however, this association did not remain statistically significant after FDR correction. No association with PFS was observed.

IFN-γ is a Th1 cytokine with an important role in cellular immune responses. The functional IFN-γ +874T/A (rs2430561) variant has been investigated in immune-mediated diseases [34] and lymphoid malignancies [7,35]. Stern et al. found no significant effect of the IFN-γ +874T/A polymorphism on lymphoma development or survival in patients with post-transplant NHL [35]. In classical HL, Ghesquières et al. evaluated IFNG rs2430561 (+874) in two independent cohorts and found no significant overall association with PFS [7]. In our study, the IFN-γ (+874) TT genotype was more frequent in patients with HL at the nominal significance level; however, this association did not remain statistically significant after FDR correction, and no association with PFS was observed.

TGF-β is an important negative regulator of lymphopoiesis and can induce apoptosis in human B cells [36], although TGF-β1 may also contribute to malignant transformation in B-cell lymphoproliferative disorders [37]. Resistance of malignant B cells to TGF-β-mediated apoptosis and increased TGF-β1 expression have been described in lymphoid malignancies [37,38]. TGF-β1 polymorphisms have been associated with an unfavorable disease course in NHL [10], and Vitiello et al. reported an increased risk of hematological malignancies associated with the TGF-β1 codon 10 TT genotype [9]. However, data specifically addressing allele-level associations of TGF-β1 codon 10 and codon 25 variants with HL susceptibility remain limited. In our cohort, the TGF-β1 codon 10 TT genotype was more frequent in patients with HL at the nominal level, although this association did not remain significant after FDR correction. More notably, the codon 10 T allele remained significantly more frequent in patients with HL after FDR adjustment, supporting a potential association with HL susceptibility. No significant genotype- or allele-level association was observed for TGF-β1 codon 25. The predefined multilocus genotype group comprising TC/GC, CC/GG, and TT/GC combinations was also more frequent in patients with HL at the nominal level, but this association did not persist after FDR correction. None of the evaluated TGF-β1 variants were significantly associated with PFS.

Established prognostic factors in HL include disease stage, age, sex, B symptoms, anemia, leukocytosis, lymphopenia, albumin, ESR, and β2-microglobulin [15,39,40,41]. In our cohort, bone marrow involvement remained associated with inferior PFS in the multivariable Firth penalized Cox model; however, the small number of affected patients and the wide confidence interval indicate considerable uncertainty in the magnitude of this association. Among the cytokine polymorphisms, most variants were not associated with PFS. The IL-6 (−174) CC genotype showed an association with inferior PFS, but this finding, based on only two carriers, should be regarded as exploratory and requires confirmation in larger cohorts. These findings suggest that genetic variants associated with HL susceptibility may not necessarily have the same relevance for subsequent clinical outcomes.

The main limitations of this study include its retrospective design, relatively small sample size, relatively short median follow-up, and the multiple genetic and clinical comparisons performed. Although false discovery rate (FDR) correction was applied to the association analyses, the possibility of chance findings cannot be completely excluded, particularly given the limited sample size and sparse subgroups. The retrospective assembly of the cohort over a long clinical period may also have introduced selection bias. Sparse subgroups, particularly the IL-6 (−174) CC genotype (*n* = 2) and bone marrow involvement (*n* = 6), limited the precision of the prognostic estimates. Although Firth penalized Cox regression was used to reduce small-sample and sparse-data bias, these findings remain uncertain and should be interpreted cautiously and validated in larger, independent cohorts.

## 5. Conclusions

Our findings suggest that selected cytokine gene variants may be associated with HL susceptibility. After FDR correction, significant allele-level associations remained for IL-6 (−174) G, TGF-β1 codon 10 T, and IL-10 (−1082) G, IL-10 (−819) C, and IL-10 (−592) C alleles. Bone marrow involvement remained associated with inferior PFS, whereas the association observed for the IL-6 (−174) CC genotype should be considered exploratory because of the very small number of carriers. Further studies in larger, independent cohorts are required to confirm these findings.

## Figures and Tables

**Table 1:** Demographic and Clinical Characteristics of Patients with Hodgkin Lymphoma.

Characteristic	*n* (%)	Median (Q1–Q3)
Gender		
Male	43 (61.4)	
Female	27 (38.6)	
Age (years)		
<45	56 (80.0)	32.5 (26.0–41.5)
≥45	14 (20.0)	
Histological subtype		
NS	40 (57.1)	
MS	27 (38.6)	
LD	1 (1.4)	
LR	2 (2.9)	
WBC (/µL)		
≤15,000	60 (85.7)	9100 (7492.5–12,897.5)
>15,000	10 (14.3)	
Lymphocytes (/µL)		
<600	3 (4.3)	1670 (1112.5–2262.5)
≥600	67 (95.7)	
Hemoglobin (g/dL)		
<10.5	11 (15.7)	12.6 (11.15–13.88)
≥10.5	59 (84.3)	
ESR (mm/h)		
<50	43 (61.4)	38 (18–67.75)
≥50	27 (38.6)	
CRP (mg/L)		
<5	14 (20.0)	20.5 (5.33–54.5)
≥5	56 (80.0)	
β2-microglobulin (mg/L)		
≤2.4	51 (72.9)	2.1 (1.70–2.58)
>2.4	19 (27.1)	
Albumin (g/dL)		
<4	35 (50.0)	3.95 (3.53–4.30)
≥4	35 (50.0)	
LDH (U/L)		
<480	67 (95.7)	234.5 (199.25–297.25)
≥480	3 (4.3)	
Ann Arbor stage		
I	6 (8.6)	
II	26 (37.1)	
III	16 (22.9)	
IV	22 (31.4)	
Stage classification		
Early stage	32 (45.7)	
Advanced stage	38 (54.3)	
Progression		
No	50 (71.4)	
Yes	20 (28.6)	
Bone marrow involvement		
No	64 (91.4)	
Yes	6 (8.6)	
Bulky disease		
No	65 (92.9)	
Yes	5 (7.1)	
EORTC risk group		
Favorable	15 (46.9)	
Unfavorable	17 (53.1)	
IPS-7		
Low	20 (52.6)	
Moderate	16 (42.1)	
High	2 (5.3)	

Abbreviations: HL, Hodgkin lymphoma; NS, nodular sclerosis; MS, mixed cellularity; LD, lymphocyte-depleted; LR, lymphocyte-rich; WBC, white blood cell count; ESR, erythrocyte sedimentation rate; CRP, C-reactive protein; LDH, lactate dehydrogenase; EORTC, European Organisation for Research and Treatment of Cancer; IPS-7, International Prognostic Score. Continuous variables are presented as median (Q1–Q3). EORTC risk stratification was applicable to patients with early-stage disease (*n* = 32), whereas IPS-7 was evaluated in patients with advanced-stage disease (*n* = 38).

**Table 2:** Comparison of Cytokine Gene Variant Frequencies between Patients with Hodgkin Lymphoma and Healthy Controls.

Polymorphism	Genotype/Allele	Control, *n* (%)	HL, *n (%)*	OR (95% CI)	Raw *p*	FDR-Adjusted *p*
TNF-α (−308)	GG	58 (82.85)	47 (67.14)	0.4230 (0.1908–0.9378)	0.051	0.1225
TNF-α (−308)	GA	10 (14.28)	23 (32.86)	2.9360 (1.2739–6.7659)	0.0160	0.1146
TNF-α (−308)	AA	2 (2.87)	0 (0.00)	0.1943 (0.0092–4.1254)	0.4964	0.5216
TNF-α (−308)	G allele	126 (90.00)	118 (84.28)	0.5959 (0.2913–1.2192)	0.2110	0.2411
TNF-α (−308)	A allele	14 (10.00)	22 (15.72)	1.6779 (0.8203–3.4329)	0.2110	0.2411
IFN-γ (+874)	TT	6 (8.57)	16 (22.86)	3.1606 (1.1558–8.6430)	0.0349	0.1147
IFN-γ (+874)	AT	38 (54.29)	33 (47.14)	0.7513 (0.3864–1.4596)	0.4989	0.5216
IFN-γ (+874)	AA	26 (37.14)	21 (30.00)	0.7252 (0.3584–1.4674)	0.4741	0.5216
IFN-γ (+874)	T allele	50 (35.71)	65 (46.43)	1.5601 (0.9662–2.5202)	0.0890	0.1187
IFN-γ (+874)	A allele	90 (64.29)	75 (53.57)	0.6410 (0.3968–1.0354)	0.0890	0.1187
IL-6 (−174)	GG	41 (58.58)	54 (77.14)	2.3872 (1.1467–4.9702)	0.0299	0.1146
IL-6 (−174)	GC	25 (35.71)	14 (20.00)	0.4500 (0.2098–0.9653)	0.0586	0.1225
IL-6 (−174)	CC	4 (5.71)	2 (2.86)	0.4852 (0.0859–2.7405)	0.6806	0.6806
IL-6 (−174)	G allele	107 (76.43)	122 (87.14)	2.0903 (1.1128–3.9262)	0.0302	0.0483 *
IL-6 (−174)	C allele	33 (23.57)	18 (12.86)	0.4785 (0.2547–0.8985)	0.0302	0.0483 *
TGF-β1 (T10/C10) (codon 10)	TT	22 (31.43)	36 (51.43)	2.3100 (1.1600–4.5998)	0.0257	0.1146
TGF-β1 (T10/C10) (codon 10)	TC	40 (57.14)	31 (44.29)	0.5963 (0.3055–1.1632)	0.1763	0.2984
TGF-β1 (T10/C10) (codon 10)	CC	8 (11.43)	3 (4.28)	0.3470 (0.0881–1.3676)	0.2076	0.2984
TGF-β1 (T10/C10) (codon 10)	T allele	84 (60.00)	103 (73.57)	1.8560 (1.1194–3.0769)	0.0224	0.0464 *
TGF-β1 (T10/C10) (codon 10)	C allele	56 (40.00)	37 (26.43)	0.5388 (0.3250–0.8937)	0.0224	0.0464 *
TGF-β1 (C25/G25) (codon 25)	GG	52 (74.29)	44 (62.86)	0.5858 (0.3168–1.2070)	0.2025	0.2984
TGF-β1 (C25/G25) (codon 25)	GC	18 (25.71)	26 (37.14)	1.7071 (0.8285–3.5170)	0.2025	0.2984
TGF-β1 (C25/G25) (codon 25)	CC	0 (0.00)	0 (0.00)	-	-	-
TGF-β1 (C25/G25) (codon 25)	G allele	122 (87.14)	114 (81.43)	0.6468 (0.3367–1.2430)	0.2502	0.2502
TGF-β1 (C25/G25) (codon 25)	C allele	18 (12.86)	26 (18.57)	1.5458 (0.8045–2.9700)	0.2502	0.2502
TGF-β1 multilocus genotype combination (codons 10/25)	TT/GG, TC/GG	52 (74.29)	44 (62.86)	0.5858 (0.2843–1.2070)	0.2025	0.3114
TGF-β1 multilocus genotype combination (codons 10/25)	TC/GC, CC/GG, TT/GC	10 (14.29)	23 (32.86)	2.9360 (1.2739–6.7659)	0.0161	0.0897
TGF-β1 multilocus genotype combination (codons 10/25)	CC/GC, CC/CC, TT/CC, TC/CC	8 (11.42)	3 (4.28)	0.3470 (0.0881–1.3676)	0.2076	0.3114
IL-10 (−1082)	GG	16 (22.86)	29 (41.43)	2.3872 (1.1469–4.9689)	0.0293	0.1146
IL-10 (−1082)	GA	28 (40.00)	23 (32.88)	0.7340 (0.3678–1.4649)	0.4826	0.5216
IL-10 (−1082)	AA	26 (37.14)	18 (25.71)	0.5858 (0.2844–1.2068)	0.2023	0.2984
IL-10 (−1082)	G allele	60 (42.85)	80 (57.14)	1.7778 (1.1073–2.8547)	0.0232	0.0464 *
IL-10 (−1082)	A allele	80 (57.14)	60 (42.85)	0.5624 (0.3504–0.9033)	0.0232	0.0464 *
IL-10 (−819)	CC	40 (57.14)	54 (77.14)	2.5310 (1.2176–5.2632)	0.0193	0.1146
IL-10 (−819)	CT	24 (34.28)	13 (18.57)	0.4371 (0.2006–0.9524)	0.0545	0.1225
IL-10 (−819)	TT	6 (8.57)	3 (4.28)	0.4776 (0.1145–1.9916)	0.4932	0.5216
IL-10 (−819)	C	104 (74.29)	121 (86.43)	2.2046 (1.1923–4.0766)	0.0156	0.0464 *
IL-10 (−819)	T	36 (25.71)	19 (13.57)	0.4537 (0.2453–0.8389)	0.0156	0.0464 *
IL-10 (−592)	CC	40 (57.14)	54 (77.14)	2.5310 (1.2176–5.2632)	0.0193	0.1146
IL-10 (−592)	CA	24 (34.28)	13 (18.57)	0.4371 (0.2006–0.9524)	0.0545	0.1225
IL-10 (−592)	AA	6 (8.57)	3 (4.28)	0.4776 (0.1145–1.9916)	0.4932	0.5216
IL-10 (−592)	C	104 (74.29)	121 (86.43)	2.2046 (1.1923–4.0766)	0.0156	0.0464 *
IL-10 (−592)	A	36 (25.71)	19 (13.57)	0.4537 (0.2453–0.8389)	0.0156	0.0464 *
IL-10 multilocus genotype combination (−1082/−819/−592)	GCC/GCC	16 (22.86)	29 (41.43)	2.4102 (1.1875–4.9702)	0.0299	0.0897
IL-10 multilocus genotype combination (−1082/−819/−592)	GCC/ACC, GCC/ATA	28 (40.00)	22 (31.43)	0.6873 (0.3429–1.3780)	0.3778	0.3778
IL-10 multilocus genotype combination (−1082/−819/−592)	ACC/ACC, ACC/ATA, ATA/ATA	26 (37.14)	19 (27.14)	0.6305 (0.3081–1.2902)	0.2776	0.3331

Abbreviations: HL, Hodgkin lymphoma; OR, odds ratio; CI, confidence interval; TNF-α, tumor necrosis factor-alpha; IFN-γ, interferon-gamma; IL, interleukin; TGF-β1, transforming growth factor-beta 1; FDR, false discovery rate. Raw *p*-values and Benjamini–Hochberg FDR-adjusted *p*-values are reported. FDR correction was applied within three predefined hypothesis families: genotype comparisons (23 tests), allele-frequency comparisons (8 locus-level tests; identical adjusted *p*-values are shown for complementary alleles), and predefined multilocus genotype-combination comparisons (6 tests). ORs were calculated with HL as the outcome; OR > 1 indicates a positive association with HL, whereas OR < 1 indicates an inverse association. Multilocus genotype combinations were predefined from PCR-SSP patterns using the manufacturer-provided evaluation form and were not computationally inferred haplotypes. For TGF-β1 combinations, the notation indicates the codon 10 genotype followed by the codon 25 genotype (e.g., TC/GC). * FDR-adjusted *p* < 0.05 was considered statistically significant.

**Table 3:** Univariate Log-Rank Analysis of Clinical Prognostic Factors and Cytokine Gene Polymorphisms for Progression-Free Survival.

Variable	Category	PFS Events, *n* (%)	Log-Rank χ^2^	*p*
Sex	Male (*n* = 43)	12 (27.9)	0.762	0.3828
	Female (*n* = 27)	8 (29.6)		
Age	<45 (*n* = 56)	16 (28.6)	0.158	0.6912
	≥45 (*n* = 14)	4 (28.6)		
WBC (/µL)	≤15,000 (*n* = 60)	19 (31.7)	1.522	0.2174
	>15,000 (*n* = 10)	1 (10.0)		
Hb (g/dL)	<10.5 (*n* = 11)	4 (36.4)	2.257	0.1330
	≥10.5 (*n* = 59)	16 (27.1)		
ESR (mm/h)	<50 (*n* = 43)	14 (32.6)	0.021	0.8852
	≥50 (*n* = 27)	6 (22.2)		
CRP (mg/L)	<5 (*n* = 14)	3 (21.4)	1.493	0.2217
	≥5 (*n* = 56)	17 (30.4)		
β2-MG (mg/L)	≤2.4 (*n* = 51)	15 (29.4)	0.046	0.8299
	>2.4 (*n* = 19)	5 (26.3)		
Alb (g/dL)	<4 (*n* = 35)	11 (31.4)	3.663	0.0556
	≥4 (*n* = 35)	9 (25.7)		
LDH (U/L)	<480 (*n* = 67)	18 (26.9)	0.359	0.5492
	≥480 (*n* = 3)	2 (66.7)		
Stage	Early stage (*n* = 32)	9 (28.1)	0.419	0.5175
	Advanced stage (*n* = 38)	11 (28.9)		
EORTC	Favorable risk (*n* = 15)	5 (33.3)	2.187	0.1391
	Unfavorable risk (*n* = 17)	4 (23.5)		
Bone marrow involvement	Absent (*n* = 64)	15 (23.4)	24.866	<0.001 *
	Present (*n* = 6)	5 (83.3)		
TNF-α −308	GG (*n* = 47)	15 (31.9)	0.838	0.3599
	GA/AA (*n* = 23)	5 (21.7)		
IL-6 −174	CC (*n* = 2)	2 (100.0)	4.803	0.0284 *
	GG/GC (*n* = 68)	18 (26.5)		
IFN-γ +874	AT/AA (*n* = 54)	14 (25.9)	0.208	0.6486
	TT (*n* = 16)	6 (37.5)		
TGF-β1 codon 25	CC + GC (*n* = 26)	8 (30.8)	0.027	0.8693
	GG (*n* = 44)	12 (27.3)		
TGF-β1 codon 10	CC (*n* = 3)	2 (66.7)	2.248	0.1338
	TT/TC (*n* = 67)	18 (26.9)		
IL-10 −1082	AA (*n* = 18)	4 (22.2)	1.032	0.5970
	GA (*n* = 23)	8 (34.8)		
	GG (*n* = 29)	8 (27.6)		
IL-10 −819	CC (*n* = 54)	16 (29.6)	1.511	0.4697
	CT (*n* = 13)	4 (30.8)		
	TT (*n* = 3)	0 (0.0)		
IL-10 −592	CC (*n* = 54)	16 (29.6)	1.511	0.4697
	CA (*n* = 13)	4 (30.8)		
	AA (*n* = 3)	0 (0.0)		
IL-10 multilocus genotype combination	Other multilocus combinations (*n* = 41)	12 (29.3)	0.027	0.8705
	GCC/GCC (*n* = 29)	8 (27.6)		

Abbreviations: PFS, progression-free survival; WBC, white blood cell count; Hb, hemoglobin; ESR, erythrocyte sedimentation rate; CRP, C-reactive protein; β2-MG, beta-2 microglobulin; Alb, albumin; LDH, lactate dehydrogenase; EORTC, European Organisation for Research and Treatment of Cancer. PFS was estimated using the Kaplan–Meier method, and differences between groups were compared using the log-rank test. PFS events are presented as *n* (%) within each category. The EORTC analysis included early-stage patients only (*n* = 32). For variables with more than two categories, the *p*-value represents the overall log-rank test. All tests were two-sided, and * *p* < 0.05 was considered statistically significant.

**Table 4:** Multivariable Firth Penalized Cox Regression Analysis of Progression-Free Survival.

Variable	HR	95% CI	*p*
Bone marrow involvement (reference: absent)	11.62	3.61–34.66	<0.001 *
IL-6 (−174) CC genotype (reference: GG/GC)	7.09	1.34–25.29	0.025 *

Abbreviations: HR, hazard ratio; CI, confidence interval; PFS, progression-free survival. The estimates should be interpreted cautiously because bone marrow involvement was present in only six patients and the IL-6 (−174) CC genotype was present in only two carriers. * *p* < 0.05.

## Data Availability

The datasets generated and/or analyzed during the current study are available from the corresponding author upon reasonable request.
